# Prevalence, pathogenic bacteria, and risk factors associated with pediatric sepsis among under five children in a rural district hospital in Rwanda

**DOI:** 10.1371/journal.pone.0327425

**Published:** 2025-06-27

**Authors:** Patrick Orikiriza, Deogratius Ruhangaza, David S. Ayebare, Ezechiel Bizimana, Jean Baptiste Niyibizi, Irene Nshimiyimana, Louis Mujyuwisha, Abebe Bekele

**Affiliations:** 1 Division of Basic Medical Sciences, School of Medicine, University of Global Health Equity, Kigali, Rwanda; 2 Department of Pathology, Butaro District Hospital, Burera, Rwanda; 3 Department of Public Health, Mbarara University of Science and Technology, Mbarara, Uganda; 4 Department of Pediatrics, Inshuti Mu Buzima/ Partner’s in Health, Kigali, Rwanda; Shiraz University of Medical Sciences, IRAN, ISLAMIC REPUBLIC OF

## Abstract

**Background:**

Pediatric sepsis poses a significant health challenge in Sub-Saharan Africa, with limited data on prevalence and pathogen profiles. This study determined the prevalence of pediatric sepsis, identified bacterial pathogens, and evaluated associated risk factors among children aged 1–59 months at Butaro Hospital, Rwanda.

**Methods:**

A cross-sectional study was conducted from March 2022 to December 2022. The study included 114 children aged 1–59 months with suspected sepsis admitted to the pediatric ward at Butaro Hospital. Blood cultures were analyzed, and risk factors assessed using multiple logistic regression. Data were analyzed using Stata 17.

**Results:**

Of 114 enrolled children, 60.5% (n = 69) had positive blood cultures (95% CI: 51.2–69.1). Among these 69 children, the majority were females, **70.0%** (n = 35) (95% CI: **53.7–81.3**) and below 6 months **68.1%** (n = **15)** (95% CI: **45.3–84.7**). Pathogenic bacteria identified were Coagulase-Negative Staphylococci (CNS), 59.4% (n = 41) and *Staphylococcus aureus*, 40.6% (n = 28). Female gender (AOR = 2.4, 95% CI: 1.0–5.4, p = 0.045) and leukocytosis (AOR = 6.0, 95% CI: 2.0–20.2, p = 0.003) were the risk factors for sepsis.

**Conclusions:**

The study reveals a high prevalence of sepsis among children under-five, especially females and less than 6 months with female gender and diagnosis with leukocytosis being a significant risk factor. Diagnostic strategies should focus on WBC counts to better manage at-risk children. These single-center study results however may not be broadly representative due to regional and resource differences, but they offer valuable insights for improving pediatric care in similar low-resource settings.

## Introduction

Pediatric sepsis represents a significant global health challenge, with an estimated 2.9 million cases occurring annually among children under five years old [1] and 21.7% [2]. The prevalence of sepsis in this age group can range from 12% to 20% in hospital settings within low- and middle-income countries [3]. One of the major challenges is the lack of adequate capacity to recognize and diagnose this clinical condition [4]. In low resource settings, the lack of these universally acceptable definitions, standard microbiological investigations, are more pronounced, leading to reliance on nonspecific clinical signs that are often subjective [5,6]. This greatly hampers early detection and initiation of prompt interventions, leading to unfavorable outcomes.

In East Africa, most studies on sepsis have focused on neonates but with high prevalence rates [7–9]. This pressing issue is compounded by the region’s healthcare constraints. Delayed hospital visits as a result of reliance on traditional medicine practices, limited knowledge, scarcity of resources, are some of the gaps and challenges linked to poor pediatric sepsis outcomes in Tanzania [10]. A similar trend has also reported in Kenyan studies [11,12].

Although specific prevalence figures for rural Rwanda are limited, the available data suggests that the burden of sepsis is substantial and underreported in such settings [13,14].

Prior research on sepsis in Rwanda has often had limitation; mostly descriptive, concentrated on adults, majorly in urban centers or focused on broader regional data [13,15–17]. This study offers a detailed analysis within a specific rural district hospital in Rwanda. The objectives included determining the prevalence of sepsis, identifying the prevalent bacterial pathogens involved, and evaluating risk factors. This provides crucial insights into the local epidemiology of sepsis, which is essential for improving diagnosis and treatment protocols in rural and resource-limited environments.

## Materials and methods

### Study design

This cross-sectional study was conducted from 31^st^ March 2022–3^rd^ December 2022 at Butaro Hospital, a rural district hospital in Burera, Rwanda.

### Participants

This was an exploratory study leading to further research in this population. However, using a 90% confidence level and 5% margin of error and a prevalence of neonatal sepsis in Rwanda (10%), the sample size is estimated at 99 participants [18]. The study included 114 children aged 1–59 months admitted to the pediatric wards with suspected sepsis.

#### Inclusion criteria.

This was based on clinical suspicion of sepsis, defined as fever or hypothermia with a negative malaria test, according to Rwanda national clinical guidelines.

#### Exclusion criteria.

These included, older children above 15, children without a parent or guardian, refusal of consent from parent or guardian, guardians unable to understand English or Kinyarwanda, and children with severe underlying conditions unrelated to sepsis.

### Data collection

Blood samples were obtained from all participants and cultured manually in a microbiology laboratory at the University of Global Health Equity, Rwanda. Clinical data were collected, including demographics, clinical symptoms, and laboratory findings. Risk factors were analyzed using multiple logistic regression. Temperature, respiratory rate, circulatory rate, skin condition, urinary system, neurological signs, and WBC levels, among other parameters, were also assessed (supplemental questionnaire attached). Sepsis was confirmed if an isolate was obtained from two microbiology blood culture bottles.

### Statistical analysis

Data were analyzed using Stata 17 (StataCorp LLC, 2021). Descriptive statistics were used to summarize demographic and clinical characteristics of the study population. To identify potential risk factors for sepsis, univariable logistic regression was initially performed. Predictor variables included childhood and parental characteristics, contributing to sepsis outcome.

Factors with p – values <02 were subsequently included in the multiple logistic regression analysis. Variables with a significance threshold of p < 0.05 were considered statistically significant.

Blood cultures were considered positive when the same bacterial isolate was identified in two culture bottles, negative if at least one bottle showed no growth and contaminated if both were contaminated. The Rwanda national paediatric guidelines consider decreased and increased WBC count (leukocytosis) to be < 4,000 mm^3^> and 12,000mm^3^ respectively [19]. Although there were no missing data nor erroneous data deletions, we had considered these in the sample size estimation.

## Results

### Socio-demographic characteristics

A total of 114 children were included in the final analysis. The majority were under five years old, with a slightly higher proportion of males (52%) (Table 1).

**Table 1:** Demographic characteristics and clinical presentation of hospitalized children under 5 years of age.

Characteristics	n (%)
Sex	
Male	64 (56.1)
Female	50 (43.9)
Age (months) Mean ±SD	17.5 (14.5)
<6 months n (%)	22 (19.3)
6-11 months n (%)	29 (25.4)
≥ 12 months n (%)	63 (55.3)
Meningeal Symptoms	
No	107 (93.9)
Yes	7 (6.1)
Shock	
No	101 (88.6)
Yes	13 (11.4)
WBC (leukocytosis)	
Normal	84 (73.7)
High	30 (26.3)
Hospital Duration (days)	
<5 days	94 (82.5)
≥5 days	20 (17.5)
Antibiotics Usage	
Yes	90 (78.9)
No	24 (21.1)

### Prevalence and pathogens

Positive blood cultures were obtained in 60.5% (n = 69) of the cases (95% CI: 51.2–69.1). Blood cultures were considered positive when the same bacterial isolate was identified in two culture bottles. The prevalence of sepsis is higher among females, with **35 positive cases** representing **70.0%** (95% CI: **53.7–81.3**). The age group of less than 6 months shows a prevalence of **68.1%** with **15 positive cases** (95% CI: **45.3–84.7**) (Table 2).

**Table 2:** Prevalence of bacteriologically confirmed sepsis among different pediatric age groups (N = 114).

Prevalence	Positive, n (%)	95%CI
Overall	69(60.5)	51.2-69.1
Sex		
Males	34(53.1)	40.7-65.2
Females	35(70.0)	53.7-81.3
Age (months)		
<6 months	15(68.1)	45.3-84.7
6-11 months	11(62.1)	42.7-78.2
≥ 12 months	36(57.1)	44.5-68.9

The predominant pathogens identified were Coagulase-Negative Staphylococci (CNS) in 59.4% (n = 41) and *Staphylococcus aureus* in 40.6% (n = 28).

### Risk factors

Female gender (AOR = 2.4, 95% CI: 1.0–5.4, p = 0.045) and leukocytosis (AOR = 6.3, 95% CI: 2.0–20.2, p = 0.002) were the risk factors for sepsis. Other factors such as age, gender, and clinical symptoms did not show significant associations (Table 3).

**Table 3:** Factors associated with sepsis among children under 5 years of age (N = 114).

		Negative (N = 45)	Positive (N = 69)	COR (95% CI)	P	AOR (95% CI)	P
Sex							
	Male	30 (67)	34 (49)	1		1	
	Female	15 (33)	35 (51)	2.1 (0.9–4.5)	0.069	2.4 (1.0-5.4)	0.045*
Age (months)							
	<6 months	7 (16)	15 (22)	1			
	6-11 months	11 (24)	18 (26)	0.8 (0.3–2.5)	0.651		
	≥12 months	27 (60)	36 (52)	0.6 (0.3–1.7)	0.365		
Mode of Delivery							
	VD	37 (82)	57 (83)	1			
	CSD	8 (18)	12 (17)	1.0 (0.4–2.6)	0.958		
Marital Status							
	Married	37 (82)	61 (88)	1			
	Unmarried	8 (18)	8 (12)	0.6 (0.2–1.8)	0.356		
Education Level							
	Secondary +	11 (24)	16 (23)	1			
	<Secondary	34 (76)	53 (77)	1.1 (0.4–2.6)	0.877		
ANC Visits							
	4	32 (71)	49 (71)	1			
	<4	13 (29)	20 (29)	1.0 (0.5–2.3)	0.991		
Parity							
	Primigravida	15 (33)	28 (41)	1			
	Multipara	21 (47)	31 (45)	0.8 (0.3–1.8)	0.583		
	Grandmultipara	9 (20)	10 (14)	0.6 (0.2–1.8)	0.354		
Condition at birth							
	No	43 (96)	66 (96)	1			
	Yes	2 (4)	3 (4)	1.0 (0.2–6.1)	0.980		
Alcohol Intake							
	No	40 (89)	63 (91)	1			
	Yes	5 (11)	6 (9)	0.8 (0.2–2.7)	0.670		
Respiratory Distress							
	No	29 (64)	37 (54)	1			
	Yes	16 (36)	32 (46)	1.6 (0.7–3.4)	0.254		
Meningeal							
	No	44 (98)	63 (91)	1		1	
	Yes	1 (2)	6 (9)	4.2 (0.5–36.0)	0.192	7.0 (0.8- 63.5)	0.084
Shock							
	No	40 (89)	61 (88)	1			
	Yes	5 (11)	8 (12)	1.0 (0.3–3.4)	0.936		
WBC Count							
	Normal	41 (91)	44 (64)	1		1	
	Elevated	4 (9)	25 (36)	5.8 (1.9–18.2)	0.002*	6.3 (2.0-20.2)	0.002**
Hospital Stay							
	<5 days	35 (78)	59 (86)	1			
	>4 days	10 (22)	10 (14)	0.6 (0.2–1.6)	0.292		
Antibiotics							
	Yes	38 (84)	52 (75)	1			
	No	7 (16)	17 (25)	1.4 (0.9–2.1)	0.249		

*p < 0.05 **p < 0.01.

## Discussion

Our study found a high prevalence of sepsis among children under five years, especially females (70.0%) and in infants under 6 months (68.1%). This is consistent with a systematic review of neonatal sepsis in Ethiopia, which reported early onset sepsis ranging from 65% at SRH, Oromia, to 88% at Gondar Teaching Hospital, Amhara [20]. Our study’s pooled prevalence of 75.4% for early onset sepsis within the first four weeks of life aligns with these findings. While both studies indicate high prevalence rates, our study provides detailed insights into gender and age-related patterns, whereas the review offers a broader regional perspective.

Within the East African region, most studies have generally focused on neonatal sepsis, revealing its high prevalence, and emphasizing substantial challenges and gaps in the broader management of pediatric sepsis [7–11,21].

In contrast, Niyoyita et al. (2024) reported a low prevalence (12.8%) of neonatal sepsis among 422 neonates in Rwanda [18]. Although both studies highlight a higher prevalence in females, our study reports a markedly higher rate (70.0%) compared to the 13.59% observed in that study [18]. Additionally, our study emphasizes overall prevalence and age-related patterns, highlighting that sepsis is significantly associated with a neonatal age of three days or less. These findings underscore the differences in prevalence rates across pediatric age groups and the variation in risk factors among the studies. Our findings highlight the need for targeted screening and treatment in high-risk groups, emphasizing pathogen-specific strategies and age-related interventions to improve pediatric sepsis outcomes.

Coagulase-Negative Staphylococci (CNS) were the most common bacterial pathogens, consistent with findings from other studies in similar settings. Our study found a 60.5% prevalence of positive blood cultures for pediatric sepsis, notably higher than the 28.6% prevalence reported by Okube and Komen (2020) at national referral hospital, in Kenya [22]. While both studies identify higher rates among females and younger infants, our findings suggest a more severe burden of sepsis in a rural setting of Butaro.

The presence of CNS in blood cultures has been a subject of debate, raising questions about whether it indicates true infection or contamination from skin flora. However, several studies involving children and neonates have increasingly linked CNS to clinical disease manifestations [23–25]. The emergence of CNS as significant contributors to neonatal and pediatric sepsis in East Africa highlights their dual role as both true pathogens and potential contaminants in blood culture diagnostics and underscores the importance of not overlooking their clinical significance.

*Staphylococcus aureus* was also highly prevalent in our study. Studies in Rwanda have previously reported *Staphylococcus aureus* as a common cause of bloodstream infections [26,27], with similar rates also across other countries [28,29]. A study conducted in Tanzania identified *Staphylococcus aureus* as a major contributor to mortality, accounting for 40% of neonatal deaths attributed to sepsis [30], emphasizing its significant impact.

Conversely, this finding contrasts with reports from South Africa, where Gram-negative infections have increased significantly, surpassing Gram-positive pathogens as the leading cause of pediatric bloodstream and cerebrospinal fluid infections [31]. A related systematic review across African studies found that Gram-negative bacteria were responsible for 63.9% of pediatric bacteremia cases, while *Staphylococcus aureus* was reported with lower prevalence [32]. These contrasting findings further underscore the regional and global diversity in pathogen profiles.

This cross-sectional study indicated that being female and diagnosis leukocytosis predicted likelihood of sepsis risk underscores the importance of this laboratory marker in identifying at-risk children. Despite limitations such as the single-center design and potential biases, the findings provide valuable insights into local sepsis epidemiology. Increase of total WBC count is indicative of inflammation and infection [33,34]. Our study in Rwanda found female gender to be a significant risk factor for sepsis (AOR = 2.4, 95% CI: 1.0–5.4, p = 0.045). In contrast, the Kenyatta National Hospital study reported a higher prevalence of sepsis in females compared to males, but this difference was not statistically significant (AOR = 1.2, 95% CI: 0.6–2.2, p = 0.555) [22]. Similarly, in Rwanda females (UOR = 1.2, 95%CI: 0.7–2.0), p = 0.633 compared to males showed a higher likelihood of sepsis [18]. Diagnostic strategies in resource-limited settings should prioritize WBC count as a key marker for sepsis. Further research is needed to validate these findings and explore additional risk factors.

## Limitations

This study has several limitations that may impact the generalizability of its findings. First, the study was conducted at a single center, Butaro Hospital, a rural district hospital in Rwanda. As a single-center study, the results may not be fully representative of pediatric populations in other regions or healthcare settings, particularly those with different levels of infrastructure, resources, and patient demographics. The healthcare practices, diagnostic capabilities, and management strategies at Butaro may differ from those at urban hospitals or in countries with more advanced healthcare systems, which could influence sepsis presentation and outcomes.

Second, the study focused on children aged 1–59 months who were clinically suspected to have sepsis and excluded those with severe underlying conditions unrelated to sepsis. This narrowed sample may not fully capture the diversity of pediatric cases, and the findings may not apply to children with comorbidities or those from different age groups. Furthermore, the reliance on clinical suspicion for diagnosing suspected sepsis could introduce variability in the detection and management of cases, potentially leading to biases in the sample.

While the study aimed to assess risk factors associated with sepsis, the cross-sectional design limits the ability to establish causal relationships. The findings should therefore be interpreted with caution, particularly when attempting to apply them to broader or more diverse populations.

Nevertheless, this was the first study to perform microbiological culture in a rural hospital in Rwanda and could provide some specific insights that could guide other limited resource settings where basic laboratory infrastructure could be optimized to improve health outcomes among children.

## Conclusion

Pediatric sepsis remains a significant challenge in Rwanda, with female gender and leukocytosis being a crucial risk factor. Efforts to enhance early diagnosis and management based on WBC counts could improve outcomes for affected children, especially females and very young neonates. By identifying prevalent pathogens like Coagulase-Negative Staphylococci and *Staphylococcus aureus* and assessing the association of leukocytosis with sepsis risk, the research provides actionable insights that can guide targeted interventions and healthcare strategies in similar low-resource settings. Although these single-center study results may not be broadly representative, due to regional and resource differences, they offer valuable insights for improving pediatric care in similar low-resource settings.

## Supporting information

S1 Data Data pediatric shared. (XLSX)
